# Influence of anxiety and anesthetic vasoconstrictors upon hemodynamic parameters during dental procedures in controlled hypertensive and non-hypertensive patients

**DOI:** 10.4317/jced.57232

**Published:** 2021-02-01

**Authors:** Francisco-Javier Silvestre, Mayte Martinez-Herrera, Belén García-López, Javier Silvestre-Rangil

**Affiliations:** 1 Unit of Stomatology, Doctor Peset University Hospital, Avda. Gaspar Aguilar 90, 46017 – Valencia, Spain; 2 Department of Stomatology, Valencia University Medical and Dental School, C/ Gascó Oliag 1, 46010 – Valencia, Spain

## Abstract

**Background:**

To determine the influence of dental anxiety and the vasoconstrictor used in local anesthesia upon different hemodynamic parameters - systolic (SBP) and diastolic blood pressure (DBP), heart rate (HR) and peripheral oxygen saturation (SatO2) - during dental extraction and oral hygiene. The safety of local anesthesia with vasoconstrictor in patients with medically controlled hypertension was also assessed.

**Material and Methods:**

A total of 159 patients were divided into two groups according to the dental treatment received: tooth extraction (n = 106) and oral hygiene (n = 53). The hemodynamic parameters (SBP, DBP, HR and SatO2) were recorded throughout dental treatment. Patient anxiety was assessed using the Beck Anxiety Inventory (BAI), the Modified Corah’s Dental Anxiety Scale (MDAS) and the Hamilton test.

**Results:**

The HR increased after anesthetic infiltration with vasoconstrictor and decreased after the tooth extraction. However, HR remained stable in the oral hygiene group, in both hypertensive and non-hypertensive patients. The SatO2 values decreased after anesthetic infiltration with vasoconstrictor. These slight changes associated with the vasoconstrictor agent were observed in patients without anxiety, but not in patients with mild or moderate anxiety. Both SBP and DBP remained constant after local anesthetic infiltration with vasoconstrictor, regardless of whether the patients presented hypertension or moderate anxiety.

**Conclusions:**

The vasoconstrictor used in local anesthesia may induce a very subtle increase in HR, with no significant increase in patients who experience anxiety.

** Key words:**Tooth extraction, dental anesthesia, vasoconstrictor agents, dental anxiety, hypertension.

## Introduction

The number of medically compromised patients with cardiovascular risk is increasing as a result of aging of the population. The prevalence of hypertensive patients, particularly adults and elderly people, is consequently also increasing in dental practice (1). Hypertensive individuals, particularly those with poor blood pressure control, are particularly susceptible to hypertensive crises. These subjects present a chronically overactive sympathetic nervous system and can experience an important increase in blood pressure in response to a sudden rise in catecholamine levels (2,3).

In dental practice, a number of factors can cause cardiovascular effects, particularly during tooth extractions or oral surgery, such as the use of a vasoconstrictor drug (VC) in local anesthesia or patient anxiety. The use of VC represents an exogenous catecholamine source that adds to the rise in endogenous catecholamine associated with patient anxiety when facing dental treatment (4,5).

Previous studies have described increases in blood pressure (BP) and heart rate (HR) following the infiltration of local anesthesia with VC (6,7). Electrocardiographic (ECG) alterations have also been associated with patient anxiety in the context of dental treatment (8). However, other studies have observed no adverse effects upon the hemodynamic parameters during different dental procedures in patients with a history of cardiovascular disease (9-11).

Our group has evidenced the safety of VC use in patients without medical conditions (12) and in patients with well controlled arterial hypertension (5). However, to the best of our knowledge, no studies have examined the influence of dental anxiety together with the use of local anesthetics with VC upon the hemodynamic parameters of controlled hypertensive versus non-hypertensive patients.

Therefore, the present study was carried out to determine the influence of dental anxiety and the VC used in local anesthesia upon different hemodynamic parameters - systolic (SBP) and diastolic blood pressure (DBP), heart rate (HR) and peripheral oxygen saturation (SatO2) measured by pulsioxymetry - during two of the most common types of dental treatment (dental extraction and oral hygiene). In addition, we evaluated the safety of local anesthesia with VC in patients with medically controlled hypertension.

## Material and Methods

-Study design and subjects

A prospective observational study was carried out in Doctor Peset University Hospital (Valencia, Spain) between January 2018 and December 2018. We recruited individuals between 18-80 years of age seen in our hospital’s Outpatient Department of the Stomatology Service for treatment (tooth extraction / oral hygiene), with a view to evaluating the influence of the VC used in local anesthesia and dental anxiety upon different hemodynamic parameters. The study sample comprised patients without arterial hypertension and individuals with medically controlled hypertension diagnosed by a physician. Patients with diabetes, kidney problems or other systemic disease conditions were excluded, as were those who had consumed caffeine or smoked in the hour before treatment.

This was a human observational study structured according to the STROBE (Strengthening the Reporting of Observational Studies in Epidemiology) guidelines, and was conducted in accordance with the ethical principles of the Declaration of Helsinki referred to medical research involving human subjects. All procedures were approved by our hospital’s Ethics Committee (Ref. 82/17), and written informed consent was obtained from all participating subjects.

One group of patients underwent simple tooth extraction. The local anesthesia used was 4% articaine with epinephrine 1:200,000 as VC (Ultracain, Normon, S.A, Spain). A maximum of three carpules were used per surgical procedure. A second group of patients was subjected to oral hygiene treatment using ultrasonic instruments (Suprasson Newtron, Satelec, Acteon, Merignac, France) without the use of local anesthesia.

-Study variables

Medical history and lifestyle variables

We interviewed each participant about health-related characteristics. Data concerning drug treatment and lifestyle habits, including tooth brushing frequency, last visit to the dentist, and previous negative experiences were recorded.

Hemodynamic measurements 

Systolic (SBP) and diastolic blood pressure (DBP) (mmHg) and heart rate (HR) (bpm) were recorded with an automatic sphygmomanometer on the left arm and with the patient in the sitting position (Omron M3, Kyoto, Japan). Peripheral oxygen saturation (SatO2) was recorded with the Fingerchip PulseFoximeter pm-50® pulsioxymeter (Shenzhen Mindray Bio-Medical Electronics Co., Atlanta, GA, USA), placing the clamp on the left index finger.

We recorded SBP, DBP, HR and SatO2 at three timepoints during both types of dental treatment. In the case of tooth extraction, these parameters were recorded before the infiltration of local anesthesia (T1), three minutes after infiltration (T2), and at the end of extraction (T3). In the oral hygiene group, the parameters were recorded before starting treatment (T1), half-way through the procedure (T2), and at the end of treatment (T3).

Anxiety testing

Three specific questionnaires were used to assess patient anxiety. The Beck Anxiety Inventory (BAI) is a self-administered 21-item questionnaire with scores from 0-3 indicating increasing anxiety. The final scores can range from 0-63 points, and the total score was reported as follows: 0-7 (minimum anxiety), 8-15 (mild anxiety), 16-25 (moderate anxiety) and 26-63 (severe anxiety) (13). The Modified Corah’s Dental Anxiety Scale (MDAS) in turn is a self-administered questionnaire of 5 items, each of which have 5 possible answers that show increasing order of anxiety. The minimum score is 5 points and the maximum 25 points. The total score was reported as follows: < 9 (no anxiety / minimum anxiety), 9-12 (moderate anxiety), 13-14 (high anxiety) and ≥ 15 (severe anxiety) (14). Lastly, the Hamilton anxiety test was also used. This is a mixed-administered 14-item instrument applied by means of a interview in which the interviewer evaluates the severity of symptoms based on 5 possible answers scored from 0 = no symptoms to 4 = very severe or disabling symptoms. The total score is obtained from the sum of the partial scores of the 14 items, and can range from 0 = no anxiety to 56 = maximum anxiety. The total score was reported as follows: 0-17 (mild anxiety), 18-24 (mild-moderate anxiety) and 24-30 (moderate-severe anxiety) (15).

The BAI and MDAS questionnaires were administered before starting treatment, while the Hamilton anxiety test was applied in the interval between infiltration of the local anesthetesia and extraction, or half-way through the treatment in the case of oral hygiene, in coincidence with previous studies (16-18).

The patients were questioned about the degree of discomfort at the end of the treatment using a visual analog scale (VAS) scored from 0-10 (0 = no discomfort, 10 = maximum or unbearable discomfort) (19).

-Statistical analysis

The sample size was calculated to afford a statistical power of 85% in the detection of mean differences (f = 0.25) in variations of a parameter over time, and of 80% in the detection of differences between groups, with a confidence interval of 95%. Parametric continuous variables were expressed as the mean and standard deviation (SD), and qualitative data were expressed as percentages. The chi-squared test was used to compare proportions. Parametric continuous variables were compared between groups (tooth extraction versus oral hygiene) using the Student t-test for independent samples or the non-parametric Mann-Whitney U-test. The changes in the different hemodynamic parameters during both procedures (T1-T3) were compared in each group using the paired sample Student t-test, with the non-parametric Wilcoxon test for the comparison of tooth extraction versus oral hygiene, hypertensive versus non-hypertensive patients, and patients without anxiety versus patients with mild-moderate anxiety. Measurement of the degree of association between levels of anxiety at timepoint T1 was based on the Pearson linear correlation coefficient. The 95% confidence interval (95%CI) was calculated for all tests, and statistical significance was considered for *p*<0.05. The SPSS statistical package (SPSS Statistics Inc., Chicago, IL, USA) was used throughout.

## Results

This study analyzed a total of 159 subjects (87 men and 72 women) with a mean age of 49 ± 20 years. The participants were divided into two groups according to the dental procedure involved: dental extraction (n= 106) and oral hygiene treatment (n= 53). The medical history, lifestyle variables and parameters associated with the dental procedure are shown in Table 1. There were no significant differences between the groups in gender, but the patients in the extraction group were comparatively older (*p*= 0.033). There were more patients with controlled hypertension in the tooth extraction group than in the oral hygiene group (60.4% versus 24.5%; *p*<0.001). All the hypertensive patients were controlled – mostly with angiotensin II receptor blockers and diuretics, in combination or not with other antihypertensive agents (Table 1). The groups showed no differences in time elapsed from the last visit to the dentist or in previous negative dental treatment experiences.

**Table 1:**
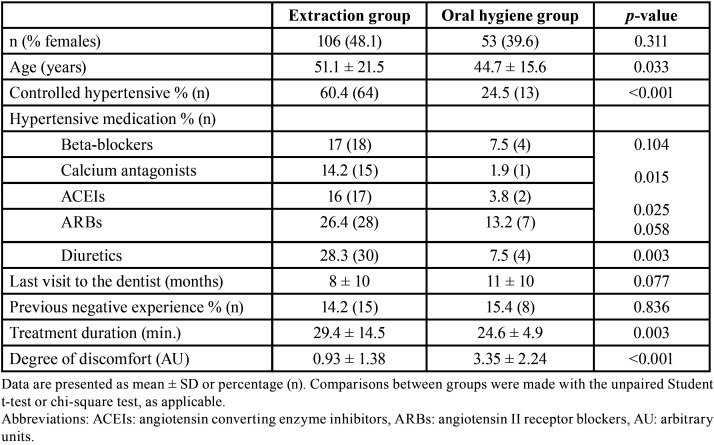
Medical history, lifestyle variables and parameters associated with the dental procedure.

Perceived discomfort after treatment (as assessed with the VAS) was comparatively greater in the oral hygiene group (where treatment was provided without local anesthesia) (*p*<0.001), while the duration of the procedure was longer in the tooth extraction group (*p*=0.003) (Table 1). The anxiety tests reflected homogeneity between the two groups, with no difference in the level of anxiety at timepoint T1 (before treatment), as evaluated with the MDAS and BAI (Table 2). Most of the participants experienced minimum or mild anxiety, with MDAS and BAI scores at T1 of 3.9 ± 5.7 and 8.3 ± 3.9 in the tooth extraction group versus 6.6 ± 9.0 and 9.7 ± 4.7 in the oral hygiene group, respectively. A strong positive correlation was found between these two anxiety tests at T1 (r=0.678; *p*<0.001), i.e., either test could be used indistinctly as an indicator in the analysis of anxiety. With regard to the Hamilton anxiety test (measured during treatment, T2), most of the patients in both groups experienced mild anxiety, though anxiety was greater among the patients undergoing oral hygiene treatment than in those subjected to tooth extraction (7.5 ± 8.3 versus 4.7 ± 5.3, respectively) (*p*=0.029).

**Table 2:**
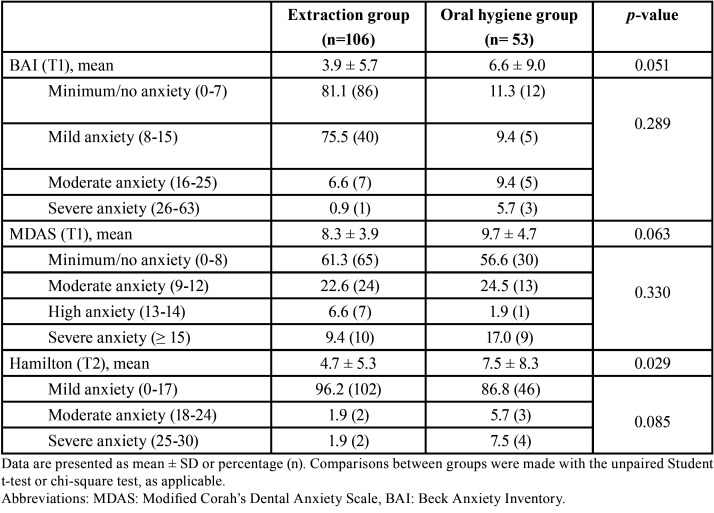
Anxiety test results in the study population.

**Figure 1:**
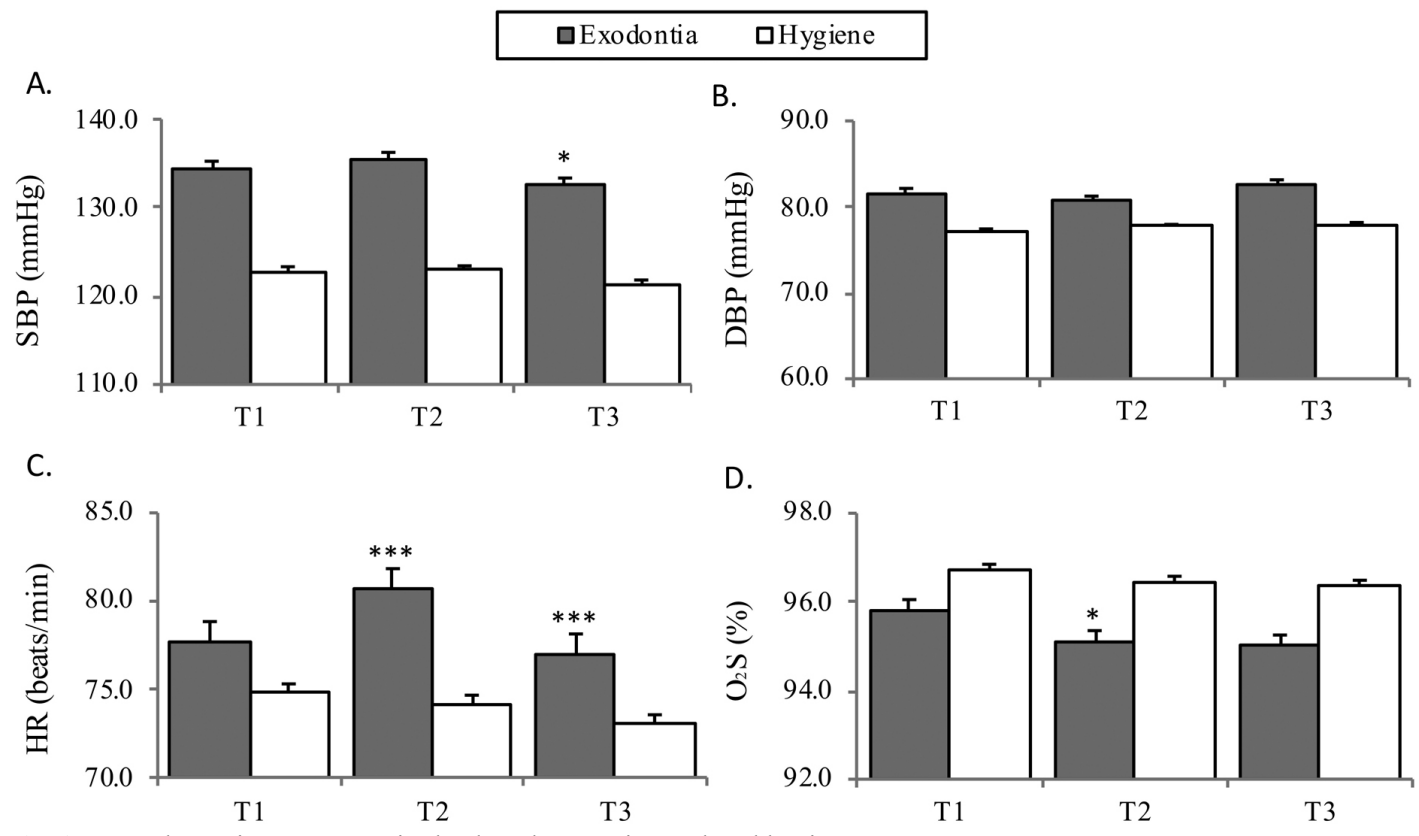
Hemodynamic parameters in the dental extraction and oral hygiene groups. Bar charts show mean + standard error. ***p≤0.001, *p<0.05 shows the changes observed during the procedure in each group, with comparison using the paired Student t-test. Abbreviations: SBP: systolic blood pressure, DBP: diastolic blood pressure, HR: heart rate, SatO2: oxygen saturation.

Regarding the analysis of the different hemodynamic parameters in the two study groups (Fig. 1), HR varied during the treatment procedure in the tooth extraction group, with a maximum value at T2 (i.e., after infiltration of the local anesthesia with VC) (*p*<0.001), and decreased after extraction at T3 (*p*<0.001). In contrast, HR remained sTable in the oral hygiene treatment group. In turn, SatO2 tended to decrease similarly in both groups in the course of treatment from T1 to T3. The decrease after anesthetic infiltration in the tooth extraction group proved statistically significant (*p*=0.011). Both SBP and DBP remained very sTable during treatment, and only a slight drop in SBP was noted at T3 in the tooth extraction group (*p*=0.044). Since there were differences in the number of hypertensive patients between the two groups, we corrected the significant changes recorded for HR between T1-T2, HR between T2-T3, SatO2 between T1-T2 and SBP between T2-T3 for the covariable hypertension using a general linear model, and found that the changes in HR remained significant between T1-T2 (*p*=0.001) and T2-T3 (*p*=0.026), regardless of whether the patients were hypertensive or not. In contrast, the change in SatO2 between T1-T2 was no longer significant (*p*=0.276), in the same way as the change in SBP between T2-T3 (*p*=0.803).

**Figure 2:**
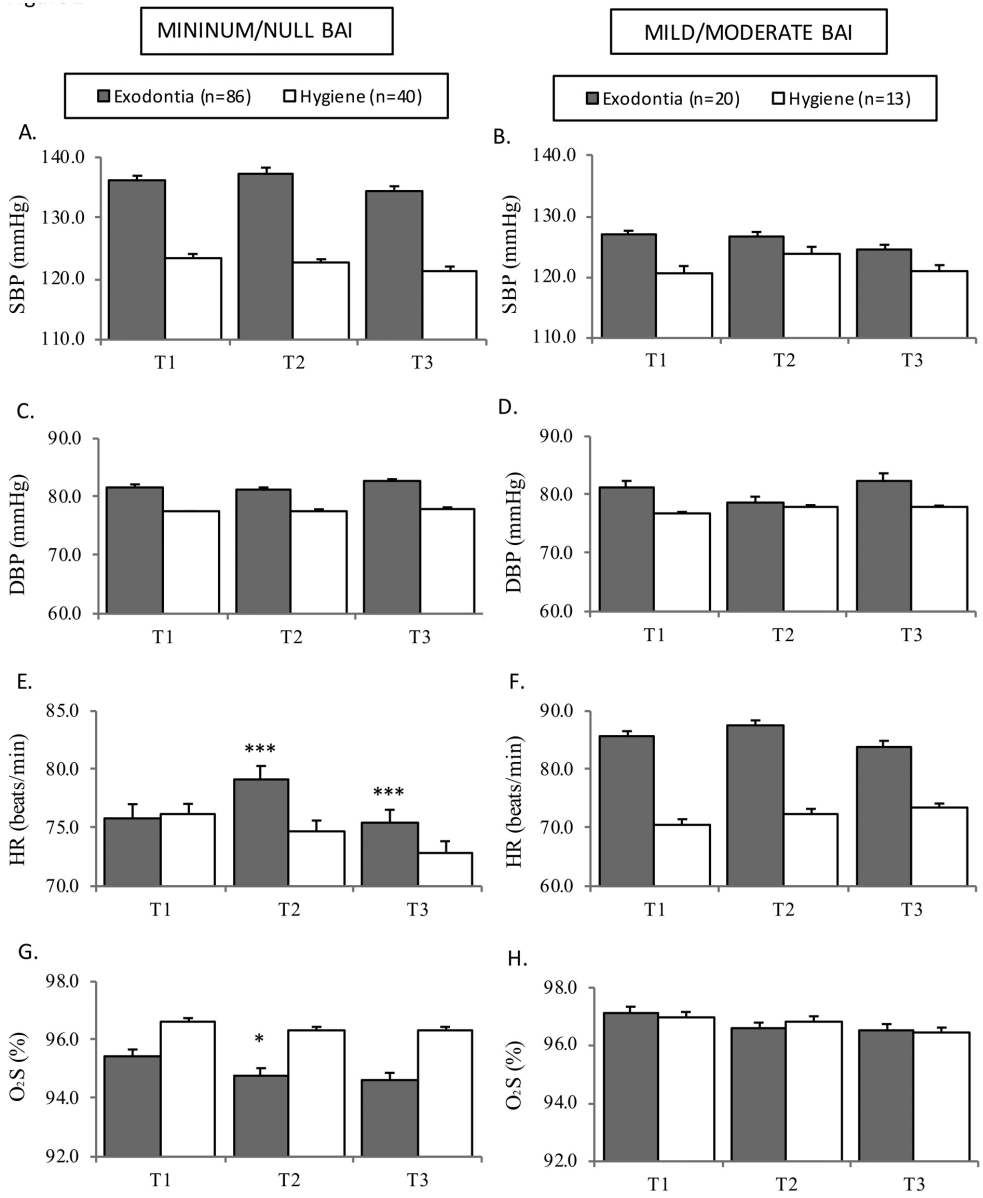
Hemodynamic parameters in the dental extraction and oral hygiene groups according to levels of anxiety. Bar charts show mean + standard error. ***p≤0.001, *p<0.05 shows the changes observed during the procedure in each group, with comparison using the paired Student t-test or Wilcoxon test. Abbreviations: SBP: systolic blood pressure, DBP: diastolic blood pressure, HR: heart rate, SatO2: oxygen saturation.

In order to analyze the influence of dental anxiety upon the hemodynamic parameters in the course of the two dental treatment procedures, we divided the oral hygiene and the tooth extraction groups into two subgroups each, according to the BAI score: no anxiety / minimum anxiety and mild-moderate or severe anxiety (Fig. 2). Only four subjects in the total sample showed severe anxiety according to the BAI (Table 2). In the subgroup of patients with no anxiety / minimum anxiety, the observed changes were the same as those seen in the global study sample (Fig. 1). HR varied significantly during treatment in the tooth extraction group, with an increase at T2 following the infiltration of local anesthetic with VC, and a decrease at T3. The values were similar in both groups at baseline (T1) (Fig. 2E). Likewise, a significant decrease was recorded in SatO2 (*p*=0.027) at T2 in the tooth extraction group (Fig. 2G). On the other hand, in the subgroup with greater anxiety as assessed by the BAI, no significant changes in HR were noted during the treatment procedure. The HR values were higher in the tooth extraction group, and remained elevated throughout treatment, with no significant increase at T2 (Fig. 2F). The SatO2 levels likewise showed no significant changes (Fig. 2H). In the oral hygiene group, HR increased slightly during treatment, though statistical significance was not reached. This was associated to anxiety, since as can be seen in Table 2, these patients experienced increased anxiety during the procedure (T2) as assessed by the Hamilton test. The rest of the hemodynamic parameters (SBP and DBP) in the tooth extraction group and also SatO2 in the oral hygiene group remained homogeneous throughout the treatment procedure, independently of the patient anxiety score.

**Figure 3:**
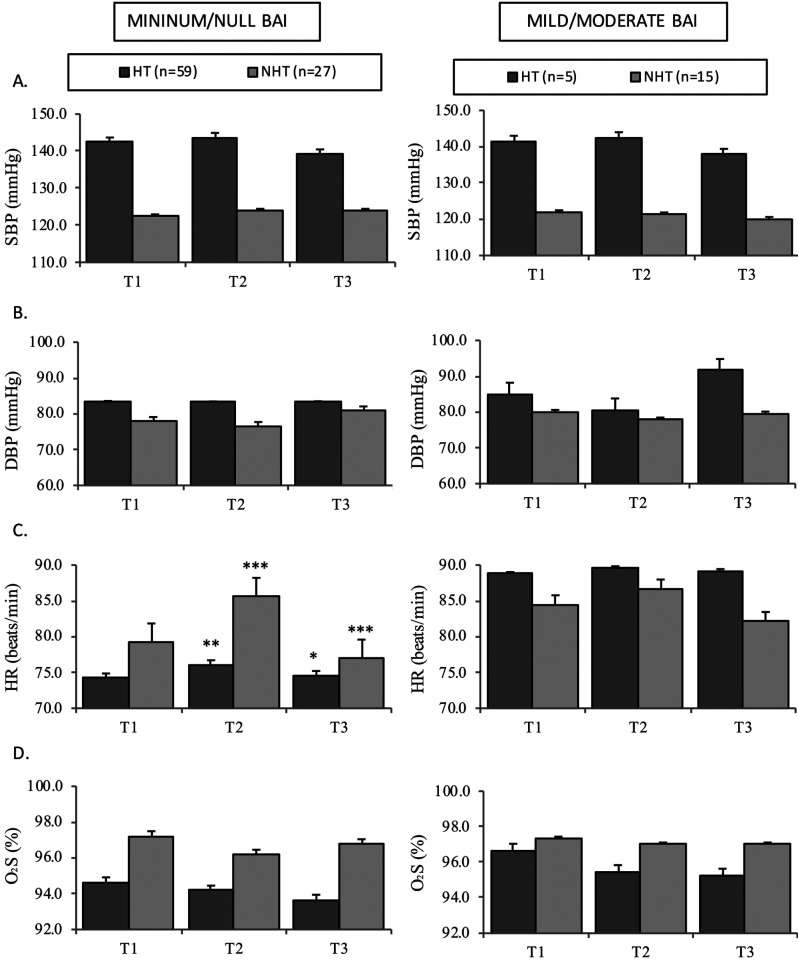
Hemodynamic parameters in the dental extraction group without anxiety, stratified into hypertensive patients and non-hypertensive subjects. Bar charts show mean + standard error. ***p≤0.001, **p<0.01, *p<0.05 shows the changes observed during the procedure in each group, with comparison using the Wilcoxon test. Abbreviations: SBP: systolic blood pressure, DBP: diastolic blood pressure, HR: heart rate, SatO2: oxygen saturation.

Lastly, in order to determine whether the use of local anesthesia with VC is safe in patients with controlled arterial hypertension, the tooth extraction group (with no anxiety / minimum anxiety, BAI < 8 points) was analyzed to compare the behavior of the hemodynamic parameters between hypertensive (n=59) and non-hypertensive individuals (n=27) (Fig. 3). We found SBP and DBP to remain sTable during the procedure in patients both with and without hypertension, though the blood pressure values were comparatively higher among those with hypertension (Fig. 3A,B). With regard to HR, an increase was noted at T2 following the infiltration of local anesthetic with VC in both groups of patients with (*p*=0.006) and without hypertension (*p*<0.001), with a significant decrease in HR at the end of tooth extraction (T3) (*p*=0.011 in patients with hypertension and *p*<0.001 in patients without hypertension) (Fig. 3C). On the other hand, SatO2 in the group of hypertensive patients was lower than in the patients without hypertension throughout the treatment procedure. Nevertheless, no significant changes in SatO2 were noted during treatment on dividing the tooth extraction patients without anxiety into hypertensive and non-hypertensive individuals (Fig. 3D).

## Discussion

The present study shows that dental anxiety and the vasoconstrictor drug used in local dental anesthesia can induce a slight increase in HR. However, the increase in HR resulting from the infiltration of local anesthetic with VC does not seem to be clinically relevant, and was moreover not seen in patients with high levels of anxiety. We therefore recommend the use of up to three carpules of local anesthetic with VC (epinephrine in proportion 1:200,000) as a safe option for the dental treatment of patients with medically controlled arterial hypertension.

Having to face dental treatment induces psychological stress in patients, even if only of minor intensity, and this in turn induces hemodynamic changes that are reflected in an increase in HR. Previous studies have described increases in HR as a result of dental anxiety (6,20,21). In line with this, we found moderate anxiety to be associated with increased HR, with values that remained elevated for the full duration of dental treatment.

Studies in which dental treatment was carried our under electrocardiographic monitoring have revealed an increased prevalence of sinus tachycardia in patients with higher levels of anxiety (8). This could be of relevance in the case of patients with previous cardiovascular disease and poor control, since the increase in adrenergic tone could result in decompensation and induce a hypertensive crisis. However, in our study we only included medically controlled hypertensive patients and moreover the individuals with dental anxiety presented mild-moderate anxiety scores. These patients showed increased HR, but the values remained stable during dental treatment and were not associated to a rise in blood pressure.

The use of VC in local anesthesia affords advantages during extraction, such as slowed systemic absorption of the anesthetic drug and lesser bleeding in the infiltrated region. Furthermore, anesthetic infiltration with VC results in increased anesthetic efficacy and less patient pain during treatment. We found patients subjected to tooth extraction with local anesthesia and VC to report less discomfort during extraction than the patients subjected to oral hygiene treatment without anesthesia. On the other hand, we found the vasoconstrictor to produce an increase in HR following infiltration of the local anesthetic, with a decrease after the completion of extraction. These variations in HR associated to VC have been observed both in patients with controlled hypertension and in individuals without hypertension, but without anxiety, and have not been seen in patients with some level of anxiety. This suggests that the increase in HR produced by VC in local anesthesia is minimal and may not be noticeable in more anxious patients where the HR remains elevated throughout the treatment procedure, and that both VC and anxiety jointly influence the observed variations in HR. On the other hand, in the tooth extraction group, the variations in HR observed among the patients with hypertension were smaller than in the patients without hypertension – a situation that could be explained by the drugs prescribed among the former. In line with our results, previous studies have shown HR to increase following the infiltration of local anesthesia, indicating a clear influence of VC, though the increase was always low and with no clinical relevance (8,22). It therefore can be affirmed that the changes in HR attributable to VC in local anesthesia are minimal, and are masked in patients with anxiety, where HR is constantly elevated to higher levels. Likewise, the use of VC did not modify the blood pressure levels in the patients subjected to tooth extraction, not even in those with medically controlled arterial hypertension. The administration of local anesthetic solutions with VC in hypertensive individuals thus seems safe, at least in small amounts (we used a maximum of three carpules per surgical procedure), since the increase in HR would be similar to that induced by anxiety alone. Consequently, and as evidenced elsewhere (23), the use of VC appears safe provided infiltration is performed correctly (slowly, avoiding intravascular injection, and using a small number of carpules).

We recorded a slight decrease in SatO2 in both dental extractions and in oral hygiene treatments, though the decrease only proved statistically significant in the tooth extraction group following infiltration of the local anesthetic with VC, and was independent of whether the patient had arterial hypertension or not. This significant decrease moreover was only seen in the subgroup patients without anxiety – not in those who were anxious to some degree. Previous studies have also evidenced a decrease in SatO2 during dental treatment (24,25). This phenomenon could be related to a degree of hypoventilation produced by prolonged patient placement in the supine position during dental treatment, or to patient air inhalation difficulties caused by the intraoral manipulations.

In addition to changes in HR and SatO2, our patients subjected to tooth extraction showed a significant decrease in SBP after extraction, in coincidence with the observations of Chaundhy *et al.* in patients with mild hypertension (26). This decrease in SBP was described as mild by Gungormus *et al*. (27) and Gedik *et al.* (28), following the infiltration of local anesthesia in periodontal treatments.

A possible limitation of our study is the fact that the patients with higher levels of anxiety were a minority within the global series. Further studies are needed, including the electrocardiographic monitoring of certain parameters to control the effects of VC and dental anxiety, particularly among patients with arterial hypertension and cardiovascular risk.

In sum, the analyzed hemodynamic parameters proved quite stable during tooth extraction and oral hygiene treatment, particularly blood pressure (both systolic and diastolic). HR increased slightly following infiltration of the local anesthetic with VC, with a slight decrease after tooth extraction. In turn, elevations in HR were associated to increased patient anxiety. We therefore suggest that HR is the most sensitive parameter for detecting the changes induced by the use of VC in local anesthesia and by the presence of anxiety during dental treatments.
